# Marfan syndrome: insights from animal models

**DOI:** 10.3389/fgene.2024.1463318

**Published:** 2025-01-06

**Authors:** Yuanyuan Jiang, Ping Jia, Xiaoying Feng, Dingding Zhang

**Affiliations:** ^1^ Marfan Research Group, Sichuan Provincial People’s Hospital, University of Electronic Science and Technology of China, Chengdu, China; ^2^ Department of Neurosurgery Nursing, Sichuan Provincial People’s Hospital, University of Electronic Science and Technology of China, Chengdu, China; ^3^ College of Medical Technology, Chengdu University of Traditional Chinese Medicine, Chengdu, China; ^4^ Sichuan Provincial Key Laboratory for Genetic Disease, Sichuan Provincial People’s Hospital, University of Electronic Science and Technology of China, Chengdu, China

**Keywords:** marfan syndrome, fibrillin-1, transforming growth factor-beta signaling pathways, animal models, gene

## Abstract

Marfan syndrome (MFS) is an inherited disorder that affects the connective tissues and mainly presents in the bones, eyes, and cardiovascular system, etc. Aortic pathology is the leading cause of death in patients with Marfan syndrome. The fibrillin-1 gene (*FBN1*) is a major gene involved in the pathogenesis of MFS. It has been shown that the aortic pathogenesis of MFS is associated with the imbalances of the transforming growth factor-beta (TGF-β) signaling pathway. However, the exact molecular mechanism of MFS is unclear. Animal models may partially mimic MFS and are vital to the study of MFS. Several species of animals have been used for MFS studies, including chicks, cattle, mice, pigs, zebrafishes, *Caenorhabditis elegans*, and rabbits. These models were developed spontaneously or in combination with genetic engineering techniques. This review is to describe the TGF-β signaling pathway in MFS and the potential application of animal models to provide new therapeutic strategies for patients with MFS.

## 1 Introduction

Marfan syndrome (MFS, OMIM: #154700) is a complex systemic connective tissue disorder that is generally inherited in an autosomal dominant manner. Aortic pathology is the primary factor causing death in patients with MFS (Lazea et al., 2021; Spencer, 2024). Multiple bone defects were first reported in 1896 by French pediatrician Antoine-Bernard and officially named MFS in 1931 (Lloyd, 1934). In McKusick. (1955) proposed that MFS is a genetic disease of connective tissue from a clinical point of view. It was not until 1986 that Sakai et al. (1986) isolated a new connective tissue protein called fibrillin-1 from human fibroblast culture medium. Fibrillin was found abundant in tissues affected in MFS patients, particularly in the aortic root, acute aortic coarctation, disproportionate growth of long bones and lens ectasia (Lazea et al., 2021). The prevalence of MFS is 20/100,000, but there isn’t any clinically confirmed MFS prevalence rates on the basis of Ghent-I or Ghent-II nosology (Groth et al., 2015). First survey of MFS prevalence in the Danish Unified Healthcare System was based on Ghent-II nosology. Groth et al. (2015) presented a prevalence of MFS of 6.5/100,000 in Denmark in 2014, and the prevalence of MFS in Denmark in 2014 is 41% higher than that of the Danish prevalence rate published almost 20 years ago. (4.6/100,000) (Fuchs, 1997). In the 1970s, the average life expectancy for MFS patients was 32 years. The introduction of aortic root replacement therapy led to a rise in the average life expectancy of MFS patients to 41 years in 1995. Today, most MFS patients can live to around 72 years with proper management (Coelho and Almeida, 2020).

This disease can frequently lead to fatal cardiovascular disease in the neonatal period or as progressively more severe cardiovascular disease in adolescence and adulthood. Veiga-Fernandez et al. (2020) demonstrated that the mortality rate within the initial 15 months of prenatal suspected cases of early-onset MFS (EOMS) was 73.68%, and the proportion of deaths in the first 5 years of postnatal confirmed patients was 61.1%. Because cardiovascular complications may not occur in early stages of MFS (Wozniak-Mielczarek et al., 2019; van Elsacker et al., 2022), early diagnosis of MFS is necessary though difficult.

Thoracic aortic disease in most patients with MFS begins as an asymptomatic enlargement of the aortic root, which gradually increases in size over time to form an aneurysm. The enlargement of the aortic aneurysm may eventually lead to acute ascending aortic dissection (called type A dissection) (Kraehenbuehl et al., 2012). Type A dissection is a life-threatening complication of MFS that can lead to a shortened life expectancy in MFS (Wang et al., 2021; Asano et al., 2022; Farag et al., 2023). Remarkably, a minority of MFS patients have type B aortic dissection. Typically, type B aortic dissection occurs without significant enlargement of the descending aorta and enlargement of the aortic root. Type B aortic dissection is less acutely fatal than in MFS patients with type A aortic dissection (Kraehenbuehl et al., 2012; Yildiz et al., 2023). Type A aortic dissection correlates with high morbidity and mortality, with the majority of people with MFS dying of complications of aortic dissection or rupture until the advent of aortic surgery, mostly before the age of 45 years ago (Silverman et al., 1994; Sharma, 2009). Presently, proper diagnosis and treatment of thoracic aortic aneurysms in patients with MFS can prevent most acute type A aortic dissection. With the development of aortic root replacement therapy since the 1970s, the life expectancy of patients with MFS approaches that of the general population (Kuzmanovic et al., 2004; Song et al., 2012; Asano et al., 2022).

In addition to thoracic aortic disease, MFS impacts other organs and tissues of the patients. Symptoms include: tall stature, disproportionately long limbs, abnormally curved spine, protruding thorax, depressed sternum, atrophied lungs, abnormally depressed acetabulum, enlarged spinal canal of the lumbar vertebral segments, and tattooing of the skin (Loeys et al., 2010; Pollock et al., 2021). Certain presentations of MFS exhibit similarities with other conditions, such as Loeys-Dietz syndrome (LDS) and Shprintzen-Goldberg syndrome (SGS) (Verstraeten et al., 2016). The clinical diagnosis of MFS requires the identification of features present throughout the system, which can be assisted by genetic testing. The MFS Ghent nomenclature was revised in 2010 to emphasize the importance of *FBN1* testing (Loeys et al., 2010).

Approximately 3,077 mutations in *FBN1* have been reported to date, with 1815 missense mutations (http://www.umd.be/FBN1/). More than 1,700 *FBN1* mutations have been identified to be potentially contributing to the development of MFS (Groth et al., 2017). The NCBI database identifies 505 organisms that are homologous to human *FBN1* (https://www.ncbi.nlm.nih.gov/gene/2200/ortholog/?scope=7776&term=FBN1), such as mouse, rat, pig and zebrafish. Using these animals with specific pathogenic variants as models is considered suitable for studying the early-onset and severe symptoms of MFS(Tae et al., 2016; de Souza et al., 2021). It is therefore critical to understand how alterations in *FBN1* lead to this multi-effect pathophysiology to determine appropriate therapies. Basic research in animal models of MFS and clinical trials of molecularly-targeted drugs have provided new therapeutic strategies for patients with MFS.

## 2 MFS related genes

In Sakai et al. (1986) proposed the diagnostic criteria for MFS. The initial set of criteria was formulated in Berlin in 1986. These criteria were established primarily to help clinicians to determine which patients should be categorized as suffering from this disease. In 1996, new diagnostic criteria called Ghent-I nosology (Ghent I) was established. *FBN1* gene was identified as a susceptibility gene for MFS (DePaepe et al., 1996). The Ghent-II nosology were modified in 2010. Loeys et al. (2010) underlined the importance of thoracic aortic disease. The revised Ghent-II nosology specifically focus on the significant overlap between Sphrintzene-Goldberg syndrome (SGS), Loeyse-Dietz syndrome (LDS), and MFS, including potentially similar involvement of bones, aortic roots, skin, and dura mater. Occasionally, SGS and vascular Ehlerse-Danlos syndrome (vEDS) overlap with that of MFS in the vascular system, dura mater, skin, and bone. The difference between MFS and related diseases is shown in Table 1. These diseases, along with non-syndromic aneurysmal syndromes, are linked to abnormal TGF-β signaling (Verstraeten et al., 2016).

**TABLE 1:** Differences between MFS and related diseases.

Disease	Gene	Chromosomal region	Features	References
Loeyse-Dietz syndrome (LDS)	TGFBR1 TGFBR1	9q22.33 3p24.1	Hypertelorism, bifid uvula or cleft palate, aortic aneurysm with tortuosity	Williams et al. (2007), Verstraeten et al. (2016)
Shprintzen-Goldberg syndrome (SGS)	FBN1 SKI	15q21.1 1p36.33-p36.32	Facial dysmorphism, marfanoid features, craniosynostosis, dolichocephaly, cardiovascular anomalies and mild to moderate mental retardation	Sood et al. (1996), Kosaki et al. (2006)
Congenital-contractural arachnodactyly (CCA)	FBN2	5q23.3	Arachnodactyly; flexion contractures of multiple joints; kyphoscoliosis; a marfanoid habitus; and abnormal “crumpled” ears. Severe CCA with cardiovascular and/or gastrointestinal anomalies	Zhang et al. (2023)
Weille-Marchesani syndrome (WMS)	FBN1 LTBP2 ADAMTS10	15q21.1 14q24.3 19p13.2	Abnormalities of the lens of the eye, short stature, brachydactyly, joint stiffness, and cardiovascular defects	Marzin et al. (2023)
Ectopia lentis syndrome	FBN1 LTBP2AD AMTSL4	15q21.1 14q24.3 1q21.2	Ectopia lentis	Cui et al. (2023)
Homocystinuria	CBS	21q22.3	Ectopia lentis and/or severe myopia, excessive height, long limbs, scoliosis, and pectus excavatum, vthromboembolism, and developmental delay/intellectual disability	Collard and Majtan (2023)
Thoracic aortic aneurysm syndrome (TAA)	TGFBR1 TGFBR1 ACTA2	9q22.33 3p24.1 10q23.31	Lack of Marfanoid skeletal features, levido reticularis, irisfloccul	Senser et al. (2021)
Arterial tortuosity syndrome (ATS)	SLC2A10	20q13.12	Elongation and tortuosity of the aorta and mid-sized arteries, focal stenosis of segments of the pulmonary arteries and/or aorta combined, soft skin, joint hypermobility, inguinal hernia, and diaphragmatic hernia. Skeletal findings include pectus excavatum or carinatum, arachnodactyly, scoliosis, knee/elbow contractures, and camptodactyly	Loeys et al. (2006)
Vascular Ehlers-Danlos Syndrome	COL3A1 COL1A2 PLOD1	2q32.2 7q21.3 1p36.22	Arterial aneurysm, dissection and rupture, bowel rupture, and rupture of the gravid uterus	Ritelli and Colombi (2020)

### 2.1 FBN1 gene in MFS

The FBN1 gene is located at 15q21.1, the cDNA is about 200 kb long and contains 65 exons with GC-rich sequences upstream of the exons (Gong et al., 2019; Lin et al., 2021). The *FBN1* precursor consists of 2,871 amino acids and contains a total of 6 structural regions *FBN1* mutations have been observed in more than 90% of the cases of MFS (Manuel Becerra-Munoz et al., 2018). Mutations in *FBN1* occur throughout most the gene. *FBN1* missense mutations account for 53%-56.1%, truncation variants 33%-36.8%, intronic variants 7.1%-13%, and total genomic rearrangements 1.8%-2.9% (Yang et al., 2018; Arnaud et al., 2021). Identification of *FBN1* genotypes for specific MFS phenotypes is complicated by the interfamilial and intrafamilial variability in the clinical features of MFS. Missense mutations in exons 24 to 32 are associated with severe EMOS (Milewicz and Duvic, 1994; Faivre et al., 2009).

### 2.2 FBN2 gene in MFS

The fibrillin-2 gene (*FBN2*, formerly known as Fib5) on chromosome 5q23-q31 is inextricably linked to fibrillin-1. The two proteins have the exact same structural domain structure as well as the same number and order of sequence motifs. At the amino acid level, structural domains B and D of fibrillin-1 and fibrillin-2 are 80% identical (Zhang et al., 1995; Dietz et al., 2005; Beene et al., 2013). Studies have demonstrated that the developmental expression of *FBN2* precedes that of *FBN1* (Robinson and Godfrey, 2000). *FBN2* is involved in the development of elastic fiber formations, while *FBN1* primarily preserves the functionality of elastic structures. *FBN2* is universally expressed in elastic tissues, but *FBN1* is primarily located in tissues that are subjected to stress and weight-bearing. Gupta *et al.* found the *FBN2* gene mutation sites in a premature infant who met the diagnostic criteria for MFS and her brother as well. Gupta et al. (2002), Gupta et al. (2004) presented MFS due to *FBN2* gene mutation in three children under 6 years in Mexico, with persistent dilatation by aortic echocardiography for more than 5 years.

### 2.3 TGFBR genes in MFS

Stheneur et al. (2008) showed that in 457 patients with MFS or related disorders the detection rates of *TGFBR1/2* mutated genes were 6.2% and 4.8% in classical MFS and 6.2% and 4.6% in incomplete classical MFS. De Cario et al. (2018) found in 75 patients with MFS that *TGFBR2* had a total of 10 polymorphisms in *TGFBR2* and 6 polymorphisms in *TGFBR1*. *TGFBR1* is located on chromosome 9q22.33 and consists of nine exons encoding the *TGFBR1* protein. Somers et al. (2016) proposed that the 6Ala allele of the *TGFBR1* could be regarded as a low-frequency variant in MFS patients. *TGFBR2* is located on chromosome 3p24.1 (Mizuguchi et al., 2004). Mutations in the *TGFBR2* are tied to MFS in individuals who do not exhibit significant ocular symptoms (Bitarafan et al., 2020). In nematode models, mutations in *TGFBR2* associated with MFS or MFS-like syndromes might cause structural perturbations in *TGFBR2*, leading to the exposure of surface structural domains, changes in subcellular localization patterns, and effect the transport of *TGFBR1* indirectly (Singh et al., 2006; Lin et al., 2019).

### 2.4 LTBP genes in MFS

The underlying TGF-β binding protein (LTBP) is a protein that targets TGF-β to the ECM by interacting with fibrillin-1 (Todorovic et al., 2005). Fibrillin is structurally related to the LTBP gene family out of which four have been identified (*LTBP1, LTBP2, LTBP3, and LTBP4*). All of these genes contain multiple tandem copies of the cb-EGF motif with two distinct 8-cysteine repeats in fibronectin and LTBP. One of these is the LTBP motif, which contains 8 cysteine residues that cluster internally in Marfan syndrome and associated microfibrillar diseases (Rifkin et al., 2018).

Sticchi et al. (2018) identified a patient with MFS in aortic symptoms of mutations in the *FBN1*, *NOTCH1*, *LTBP1*, and *TGFBR3* genes. Ramona *et al.* showed that c.1642C > T (p.Arg548*) of *LTBP2* may be associated with ocular manifestations of MFS, MVP and funnel chest. In 2010, Desir *et al.* observed that MFS might be related to mutations in the *LTBP2* gene (Desir et al., 2010). In 2019, Morlino *et al.* noted that two Romani individuals exhibited a phenotype resembling MFS due to the presence of the homozygous p.R299X variant in the *LTBP2* (Morlino et al., 2019). Bertoli-Avella *et al.* indicated that patients with *LTBP3* gene mutations exhibited elastic fiber breakage, as well as increased accumulation of collagen and proteoglycans within the aortic wall tissue (Bertoli-Avella et al., 2015). They found that individuals with mutations in this gene exhibited severe cardiovascular symptoms that closely resembled those in MFS patients. Korneva et al. (2019) detected that the absence of *LTBP3* attenuated elastic fiber breakage and focal dilatation in a mouse model of MFS lacking fibrillin-1. However, MFS mouse models with spinal deformities persisted in the absence of *LTBP3* (Zilberberg et al., 2015). MFS mice lacking *LTBP3* had increased survival and suppressed Smad2/3 and Erk1/2 activation in the aorta. Aortic aneurysms disappeared in MFS mice (Bertoli-Avella et al., 2015; Zilberberg et al., 2015). These data suggested that the latent TGF-β complex composed of *LTBP3*/TGF-β may contribute to the progression of aortic disease in MFS.

## 3 TGF-β pathway abnormalities in MFS

**FIGURE 1:**
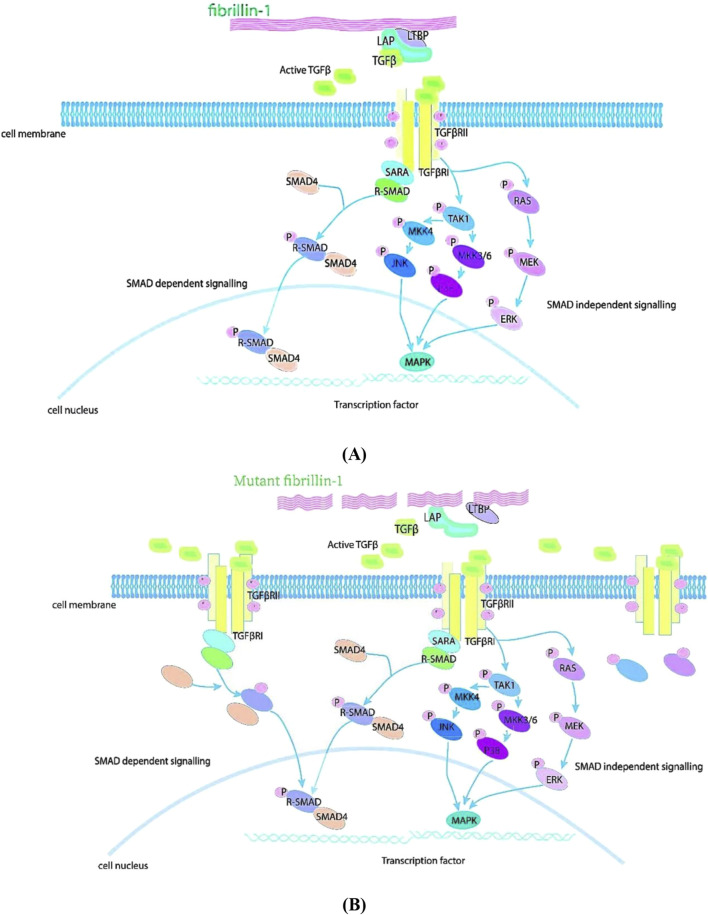
TGF-β pathway signaling in MFS. FBN1 has a role in regulating TGF-β. **(A)** FBN1 binds to a large latent complex composed of LAP, LTBP and TGF-β and regulates the concentration of activated TGF-β in the matrix. Upon activation of the complex, TGF-β binds TGF-β receptors and signals. This signal can be transmitted from the cell membrane to the nucleus via Smad-dependent or Smad-independent pathways. **(B)** Mutations in FBN1 cause the release of large amounts of active TGF-β1 from the extracellular matrix, leading to over-activation of the TGF-β signaling pathway and accelerating the destruction of the extracellular matrix.

TGF-β family play critical roles in embryonic development, adult tissue homeostasis and repair. Genetic studies in animals demonstrated that the TGF-β signaling pathway was correlated with MFS (Goumans and ten Dijke, 2018; Gensicke et al., 2020). An integral role of the TGF-β signaling pathway in the pathogenesis of MFS thoracic aortic aneurysms was identified by knocking out the *FBN1* gene in MFS model mice (Gensicke et al., 2020). FBN1 protein modulates the levels of activated TGF-β in the extracellular matrix through its interaction with a complex comprising LAP, LTBP, and TGF-β. Extracellular activation of this complex is an essential condition for the biological activity of TGF-β regulation. Upon activation of this complex, TGF-β transmits signals to two membrane-bound TGF-β receptors via costimulatory binding. Furthermore, signals are transmitted from the cell membrane to the nucleus via Smad-dependent or Smad-independent pathways (Derynck and Zhang, 2003; Shi and Massagué, 2003; Tang et al., 2018). FBN1 protein mutations causing abnormal TGF-β pathway signalling in MFS are displayed in Figure 1.

### 3.1 SMAD dependent signaling

The inactive TGF-β precursor consists of 390–442 amino acids, and the TGF-β precursor has three specialized regions consisting of an N-terminal hydrophobic signal peptide region, a 249-amino-acid latently relevant peptide region, and a C-terminal region (Shi and Massagué, 2003; Goumans and ten Dijke, 2018; Tang et al., 2018). Inactive TGF-β precursors are endopeptidase cleaved in the Golgi apparatus, and thereby forms a small latent complex. The mature TGF-β homodimer contained therein is formed by non-covalent bonding with two latent peptides and is normally secreted by the cell as a large latent complex together with LTBP. LTBP anchors inactive TGF-β to the extracellular matrix. Inactive TGF-β releases biologically active TGF-β from the latent complex through the interaction of latent-associated peptides with a variety of proteins. Upon binding of activated TGF-β to cell-surface receptors, TGF-β ligands stimulate the assembly of serine/threonine kinase complexes, which pass through the cytoplasm and protein phosphorylation initiates signal transduction (Shi and Massagué, 2003; Akhurst and Hata, 2012; Goumans and ten Dijke, 2018).

Activated TGF-βR1 specifically recognizes and phosphorylates R-Smad (receptor-regulated Smads). The R-Smad substrate activates the TGF-β receptor complex via the SMAD receptor activation anchor (SARA). Phosphorylation of R-Smad reduces its affinity for SARA and leads to the formation of a heterodimer with Smad4. Activated R-Smad forms a heterodimeric complex with Smad4. The heterodimeric complex then rapidly translocates to the nucleus. Upon entry into the nucleus, this complex interacts with transcription factors containing sequence-specific DNA-binding affinities at promoter sites to regulate gene expression. Smad4 is translocated to the nucleus only when bound to R-Smad, which can autonomously move from the cytoplasm to the nucleus in the absence of Smad4. However, when Smad4 is blocked or absent, R-Smad can translocate but lacks the ability to completely signal the nucleus through gene expression. This implies that the primary role of Smad4 is to regulate transcription rather than to transmit TGF-β signaling from the cytoplasm to the nucleus (Johnsen et al., 2002; Ten Dijke et al., 2002; Derynck and Zhang, 2003; Shi and Massagué, 2003).

### 3.2 SMAD independent signaling

In addition to Smad-independent transcription, TGF-β activates Smad independent signaling cascades. Smad independent signaling pathway includes the Erk, JNK, and p38 MAPK kinase pathways, and the mechanisms by which TGF-β activates Erk, JNK or p38 MAPK and their biological consequences remain to be elucidated. JNK and p38 MAPK signaling is activated by various MAPK kinases (MAPKKKs) in response to many stimuli. Rapid activation of Ras by TGF-β is associated with the participation of Ras in TGF-β induced Erk MAPK signaling (Derynck and Zhang, 2003; Holm et al., 2011; Mu et al., 2012). Smad independent signaling such as NO, angiotensin, WNT, NOTCH and PI3K/AKT are also related to MFS progression (Nataatmadja et al., 2013; Dong et al., 2023).

Gensicke et al. (2020) analyzed the expression levels of the atypical regulators ERK and p38 in cardiac tissues of the *FBN1*C1039G^*/+*^ MFS mouse model by Western blotting. Rouf et al. (2017) measured mouse aneurysms by thoracic echocardiography and determined levels of phosphorylated Erk1/2 (p-Erk1/2) and pSmad2 in aortic tissue. These results indicated that aneurysms in MFS model mice are linked to Erk1/2 Smad and p38 signaling. Sato et al. (2018) showed that the downstream protein of Ras, pRaf1, and pERK1/2 were significantly elevated by Western blotting. Inhibition of the Ras-induced Erk signaling pathway reduced aneurysm growth in mice. These results showed that activation of Ras/Erk MAPK signaling could induce TGF-β expression, being expected to result in aortic aneurysm growth. Chung et al. (2007) were the first ones to demonstrate that endothelial dysfunction in the thoracic aorta of patients with MFS was probably due to downregulation of NOS-induced NO production by Akt or endothelial cells. de la Fuente-Alonso et al. (2021) reported that NO overactivation of sGC-PRKG signaling induced MFS thoracic aortic disease. These pointed to the importance of NO in the progression of thoracic aortic lesions. Angiotensin II (Ang-II) directly induced aortic dilatation in a mouse model of MFS, affecting TGF-β synthesis and receptor expression. Ang-II interacts with TGF-β signaling (Yu and Jeremy, 2018). Jespersen et al. (2022) detected Notch 3 expression in aortic tissue from the *FBN1mgR/mgR* MFS mouse model and human MFS. Notch3 levels were elevated in both the MFS mouse model and human MFS. Thus, aortic abnormalities in MFS are combined with increased Notch3 activation. Granata et al. (2017) modeled the vasculature of human induced pluripotent stem cells (MFS-hipscs). Smooth muscle cells (SMCs) of MFS-hipscs origin recapitulated the pathology of Marfan’s aorta with high levels of KLF4 and P-p38, which was validated in patient samples. These results suggested that krppel-like factor 4 (KLF4) controls the p38 pathway to regulate SMC apoptosis. Activation of the mechanistic target of rapamycin (mTOR) pathway is also thought to play a role in aortic aneurysm formation in patients with MFS. The mTOR signaling pathway was shown to be significantly activated during aneurysm development, and inhibition of mTOR signaling reduced the development of aortic coarctation in animal models (Sarbassov et al., 2005; Laplante and Sabatini, 2012; Saxton and Sabatini, 2017).

## 4 Treatment of MFS

Pharmacological treatments for MFS aim to limit the rate of aortic dilatation and slow the progression of cardiovascular disease. However, MFS cannot be completely cured, and research into drug therapy remains a hot topic (Chiu, 2022).

β-blockers have been recommended as first-line therapeutic agents for MFS(Bowman et al., 2019; Deleeuw et al., 2021). β-blockers are classic drugs that selectively bind to β-adrenergic receptors, thereby antagonizing the agonistic effects of neurotransmitters and catecholamines on β receptors (Koo et al., 2017). These drugs reduce heart rate and myocardial contractility by blocking β1 and β2 receptors distributed in the heart and blood vessels, thereby reducing myocardial oxygen consumption (Fici et al., 2023). Furthermore, β-blockers inhibit the release of renin and decrease the production of angiotensin II, thus reducing systemic vascular resistance (Paolillo et al., 2021). This mechanism of action not only helps to reduce cardiac afterload and lower blood pressure, but also reduces dilatory pressure in the aortic root (Messerli et al., 2023). In patients with Marfan syndrome, dilatation of the aortic root is a serious problem that can lead to fatal aortic coarctation or rupture. β-blockers mitigate the likelihood of these complications by decelerating the rate of aortic root dilation through the above mechanisms. Several clinical trials have confirmed that β-blockers effectively reduce the rate of aortic root dilation in MFS patients (Koo et al., 2017; Jondeau et al., 2023). Although β-blockers have demonstrated efficacy in the management of Marfan syndrome, they may be associated with some side effects. Common cardiovascular adverse effects include hypotension and bradycardia, which may lead to exacerbation of heart failure in severe cases, especially in elderly patients or at high doses (Richards and Tobe, 2014; Vargas et al., 2023). It may induce or exacerbate bronchial asthma due to the blocking of β2 receptors on bronchial smooth muscle. Hypogonadism, anaphylactic rash, and gastrointestinal distress are also common side effects (Tatu et al., 2019). Therefore, the challenge of using β-blockers as a primary therapy for the prevention of aortic complications in patients with MFS is enormous.

In mice model of MFS, angiotensin receptor enkephalin inhibitors delay ascending aortic dilatation more than ARB alone (Yu and Jeremy, 2018). Thus, angiotensin receptor enkephalin inhibitor therapy may be a novel approach for the treatment of MFS (Brooke et al., 2008; Habashi et al., 2011). Angiotensin-converting enzyme inhibitors (ACEIs) reduce arterial pressure and delay atherosclerosis. Mutations in the *FBN1* gene enhance TGF-β signaling, whereas ACEIs reduce TGF-β signaling (Cavanaugh et al., 2017a). Therefore, ACEIs can be recommended for the treatment of patients with MFS. The study of animal models of MFS such as zebrafish, mice, pigs and *in vitro* models of human induced pluripotent stem cells would also help to determine the efficacy and safety of novel drugs for MFS.

“Bentall-de Bono” aortic root replacement has enhanced the lifespan of MFS patients (Ornelas-Casillas and Garcia-Arias, 2022). This surgery should be considered as early as possible when patients with MFS are labelled as high risk for ascending aortic risk factors (Dallan et al., 2021; Hlavicka et al., 2022; Ornelas-Casillas and Garcia-Arias, 2022). Lens ectasia is a common manifestation of ocular involvement in patients with MFS. Eye complications such as glaucoma and long-term blindness can be prevented by early surgical intervention. Sahay *et al.* described a microscope-guided lens aspiration technique that can be applied to treat anterior lens dislocation in children (Sahay et al., 2019). About 60% of patients with MFS have scoliosis. When the condition is severe, it can cause significant skeletal deformities, pain, and restrictive ventilatory dysfunction. It is recommended that patients with MFS may be treated surgically with symptoms (Lumban Tobing and Akbar, 2020; Rava et al., 2020). In 2017, Wang *et al.* helped an MFS couple birth a healthy newborn by using preimplantation genetic diagnosis (PGD).The PGD approach can yield healthy offspring for families at high risk for genetic disorders (Wang et al., 2017).

## 5 Animal models of MFS

At present, animal models for MFS are categorized into two groups: spontaneous animal models and experimental animal models. Animal models of MFS primarily mimic the aortic coarctation phenotype of human MFS. Although many studies have been conducted to investigate the molecular mechanisms of TGF-β and other signaling pathways in MFS with the knockout *FBN1* mouse model, there are fewer reports on other homologous animal models of MFS. The advantages and disadvantages of the MFS animal model are highlighted in Table 2.

**TABLE 2:** Advantages and disadvantages of animal models of MFS.

Animal	Technical	Outcomes	Advantage	Disadvantage	References
Chicks	Drug feeding	Death by AA	Simple operation	No human genes given	Simpson et al. (1980a)
Cattle	Multiply	AA, lordosis, elongated distal limbs, and lens ectasia	Can be passed on from one generation to the next	No human genes given	Halper (2014)
Mouse	Gene editing	fbn1*mgΔ* mouse	*mgΔ*/*mgΔ* mice showed cardiovascular abnormalities	Low extrinsic rate Cannot produce offspring	
		*mgR* mouse	*mgR/mgR* Mouse similar to MFS.	Low extrinsic rate Cannot produce offspring	Habashi et al. (2006), Cavanaugh et al. (2017b), Gharraee et al. (2022), de Souza et al. (2023), Weiss et al. (2023)
		C1039G mouse	C1039G^*/+*^ mouse similar to MFS.		
		*mgN* mouse	*mgN*/*mgN* mice showed AA	Dead within 2 weeks	
		GT-8 mouse	Heterozygous mice showed AA	Homozygous mice die early after birth	
		*H1Δ* mouse	normal growth cycles	Without aortic lesions or microfiber defects	
		*mgΔ* ^*loxpneo*^ mouse	Typical MFS phenotype		
Pigs	Gene editing	Glu433AsnfsX98	Similar to MFS	Long breeding cycle	Umeyama et al. (2016b)
Zebrafishes	Gene editing	fbn1+/-zebrafish	Similar to MFS	High fecundity and short life cycle	Yin et al. (2021)
*Caenorhabditis elegans*	Gene editing	Mua-3	MFS and MFS-like mutations in type II receptors	Little research	Fotopoulos et al. (2015b)
Rabbits	Gene editing	FBN1 Het rabbits	Typical features of MPL syndrome	Small sample size	Chen et al. (2018)

### 5.1 Spontaneous animal models

#### 5.1.1 Cattle model of MFS

Bovine MFS is an inherited disease with many of the clinical and pathologic manifestations of human MFS. The main manifestations are lens ectasia, aortic dilatation, aneurysms and ruptures. In 1990, Besser *et al.* described a phenotypically normal purebred bull that produced 72 calves from 41 females. Seven of these calves were infected. Clinical examination of siblings of infected calves revealed no skeletal, ocular or cardiovascular lesions. Neonatal calves affected are known as bovine MFS(BMFS), which suffered from scoliosis, distal limb overgrowth, and lens ectasia. These symptoms were phenotypically similar to human MFS (Besser et al., 1990). In 1994, Potter, K A and T E Besser tracked 10 BMFS cattle. Autopsy results showed that six BMFS cattle died of severe cardiovascular lesions (Potter and Besser, 1994). Morphological and ultrastructural revealed severe degenerative elastic fiber disease in the aortic tissue of BMFS. BMFS cattle with vvg-staining of sections displayed an increased ultrastructural density of elastic fibers in the skin, lungs and neck, and an increase in peripheral microprogenic fibers.

Cardiovascular lesions in BMFS are close to human MFS visually and under microscope. Therefore, BMFS can be a valuable model for exploring the molecular pathogenesis and therapeutic approaches to human MFS. However, BMFS are costly and have long breeding cycles. BMFS is rarely studied with MFS at present.

### 5.2 Experimental animal models

#### 5.2.1 Chicks model of MFS

In 1980, Simpson et al. (1980b) observed aneurysms in patients with MFS, chicks fed a copper-deficient diet and turkeys fed b-aminopropionitrile (BAPN) by light and electron microscopy. Broken elastic fibers and atrophied smooth muscle cells in the abdominal aorta were detected by Orcein-Van Gieson staining of sections and electron microscopy. The results indicated similar aneurysm phenotypes in chicks and humans. Nevertheless, this MFS-like Chicks model has not been widely used ever since.

#### 5.2.2 Mouse model of MFS

Most animal models of MFS have been created by gene editing mice. The mice models include *mgΔ*, *mgR*, C1039G, *mgN*, *H1Δ*, and *mgΔ* ^*loxpneo*^ mice.

##### 5.2.2.1 *MgΔ* mice

In Pereira et al. (1997) established the *FBN1mgΔ* mice model. In a C57BL/6J mice background, exons 19–24 of the 6 kb long *FBN1* were replaced with a neomycin-resistant expression cassette (neoR) using gene editing techniques. Expression analysis showed that the transcript level of the *mgΔ* allele was 90% lower than that of the normal *FBN1* allele. Heterozygous *mgΔ* ^*/+*^ mice had normal life expectancy, but all *mgΔ*/*mgΔ* mice died around 3 weeks after birth. *FBN1mgΔ* ^*/+*^ animals were histologically indistinguishable from wild-type mice. *FBN1mgΔ*/*mgΔ* mice had normal bones, but all suffered from cardiovascular complications, such as AA/AD. The transcript level of the *mgΔ* allele was 90% lower than that of the normal *FBN1* allele.

The neoR box sequence possibly interferes with the expression of the mutant allele, thus limits the dominant-negative effect of the mutation. Targeting experiments in mice suggest that the *FBN1* protein is primarily involved in tissue homeostasis.

##### 5.2.2.2 *MgR* mice

In Pereira et al. (1999) used gene editing in a C57BL/6 mouse background to clone the target *mgR* allele from the PGKneo cassette into the region of exons 18 and 19 and replaced exons 19 to 24 with the neomycin (neo) gene. Both mice with heterozygous and pure mutants of *mgR* were born in the anticipated proportions and did not exhibit any abnormal characteristics at birth. The *mgR/mgR* mice exhibited clinically significant scoliosis and increased thoracodorsal-ventral diameters and died of aortic coarctation in early adulthood compared to *mgR* ^*/+*^ mice (Pereira et al., 1999). Similar Gene-targeted mutant *mgR* mice with low expression of *FBN1* resulted in MFS-like manifestations. Histopathological analysis of aortic specimens demonstrated the presence of medial calcification, inflammatory fibroproliferative response, and inflammation-mediated lysis of the entrapped aneurysm.

##### 5.2.2.3 C1039G mice

In Judge et al. (2004) overexpressed an *FBN1* (C1663R) mutant in a normal mouse background using yeast artificial chromosome transgenesis. The C1039G mutation was localized to exon 25 of the cb-EGF-like structural domain encoded by the mouse *FBN1* gene (Judge et al., 2004). The *FBN1*C1039G/C1039G mice often died perinatally from vascular anomalies. The *FBN1*C1039G*/+* mice were under a normal life cycle with abnormalities of the aorta and typical skeletal abnormalities (Judge et al., 2004). Heterozygous mice with characteristics similar to MFS in humans exhibit impaired microfibre deposition, skeletal malformations and progressive deterioration of aortic wall structure.

##### 5.2.2.4 *MgN* mice

In Carta et al. (2006) created the *FBN1* gene deletion (*mgN*) mouse model. An exon of the *FBN1* gene containing an ATG codon and signal peptide coding sequence of approximately 700 bp was replaced with a phosphoglycerol kinase neo cassette and an alkaline phosphatase gene containing an internal ribosomal entry site. *mgN* ^*/+*^ mice had a normal life cycle and phenotype. However, *mgN*/*mgN* mice died within 2 weeks of birth from ruptured aortic aneurysms, impaired lung function, and/or diaphragmatic collapse. Deletion of one or both *FBN1* alleles in the context of *FBN1* deletion resulted in embryonic death. This suggests that *FBN1* is critical for substrate assembly and the initiation of embryonic development.

##### 5.2.2.5 *H1Δ* mice

In 2010, two novel knock-in mice were born. Charbonneau *et al.* performed an experiment involving C57Bl/6 mice with Cre recombinase inserted into the Rosa 26 locus, which was mated with *FBN1* mice tagged with an enhanced green fluorescent protein (eGFP). The specific lox locus was integrated into the targeting vector and the lox66 and lox77 motifs were reversed by cre-mediated recombination. eGFP coding sequence was inverted into the frame after exon 32. cbEGF region was truncated. *FBN1* was tagged with eGFP. A mice strain named GT-8 was successfully established. GT-8 homozygous mice died early after birth. Heterozygous mice had abnormal skin and dilated aorta but normal growth cycles (Charbonneau et al., 2010). Another mice model called *H1Δ* (heterozygous 1 structural domain deletion) was created through cre-mediated removal of FBN1 exon 7 (bordered by loxP sites) in a second generation of mice on a C57Bl/6 background. *H1Δ* mice had a normal growth cycle and no aortopathy or microfibrillar defects.

##### 5.2.2.6 MgΔ^loxpneo^ mice

In Lima et al. (2010) modified the neoR (*mgΔ*loxpneo) of the *mgΔ* mouse model with lox-P sequences within the context of the C57BL/6 and 129/Sv animal models by gene editing. *mgΔ* ^*loxpneo*^ from the C57BL/6 and 129/Sv backgrounds were mated with CD1 females to produce the F1 generation. Heterozygous *mgΔ* ^*loxpneo*^ of the F1 generation displayed no significant phenotype. Subsequently, the heterozygotes of the F1 generation were hybridized. The heterozygous *mgΔ* ^*loxpneo*^ mice all exhibited typical MFS phenotypes, including aortic, skeletal (primarily retroconvex), and respiratory (emphysema) phenotypes. Both crosses had normal lifespan and reproduction (Lima et al., 2010). The phenotype of the MFS model mice established by the gene editing techniques described above was dominated by aortic lesions. Clinical variants in the mice indicated that epigenetic factors were associated with disease severity.The overall expression level of *FBN1* was strongly negatively correlated with the severity of the phenotype, and thisconfirmed the relevant role of mutated *FBN1* in the pathogenesis of MFS.

#### 5.2.3 Pigs model of MFS

The advancement of reproductive biology led to genetically engineered pigs as models for human genetic diseases. Although several models of monogenic diseases exist, replication of human diseases caused by haploinsufficiency remains a major challenge. Investigating the gene regulatory mechanisms associated with haploinsufficiency can be of great practical value in establishing reliable pig models of MFS.

In Umeyama et al. (2016a) created an *FBN1* mutant (*fbn1* ^*mut*^) clone pig (+/Glu433AsnfsX98) by using genome editing and somatic cell nuclear transplantation. This model exhibited phenotypes similar to human MFS symptoms, as scoliosis, funnel chest, and aortic anomalies. Second generation (G2) pure mutant pigs exhibited typical MFS symptoms and survived for up to 28 days (Umeyama et al., 2016a). In 2022, Jack *et al.* established the *fbn1* ^*mut/+*^ (Glu433AsnfsX98/WT) porcine model by somatic cell nuclear transfer (SCNT) technique. The first method involved transferring blastocyst-stage SCNT embryos into recipient reserve sows after 5 days of long-term culture. The symptoms of MFS related to scoliosis, concave chest, delayed epiphyseal mineralization and aortic wall elastic fibre abnormalities were demonstrated in 4 of 8 *fbn1* ^*mut/+*^ pigs. Many of these symptoms were observed in the neonatal period. The second method of cloning pigs were SCNT embryos transplanted at the early cleavage stage without prolonged culture. There were two pigs that had no abnormalities in the neonatal period and developed symptoms at maturity. *In vitro* manipulation of embryos (including culture) can epigenetically alter gene expression. In the fourth generation of cloned *fbn1* ^*mut/+*^ male pigs, MFS symptoms were observed in G1 ∼ G4 stage pigs at 662 days postnatal age. Cardiovascular lesions were the predominant symptom in individuals in the G2 ∼ G4 stages. The *FBN1*Glu433AsnfsX98/WT genotype were characterized by a later onset of MFS. The phenotypic diversity and neonatal morbidity observed in G0 cloned animals appeared to decrease after G1 (Jack et al., 2022b). The *fbn1* ^*mut/+*^ profile recapitulated the variable and delayed pathogenesis of MFS. However, predicting and controlling the kinetics of symptom onset in porcine models of MFS still remains a major challenge for future investigations.

#### 5.2.4 Zebrafishes model of MFS

Zebrafish models have the advantage of clear embryonic optics, high fecundity, low rearing costs, short life cycles, ease of experimental manipulation and the fact that approximately 70% of human genes have functional homologues in zebrafish (Fontana et al., 2018).

In Chen et al. (2006) produced knockout embryos by injecting 2 independent splice-site morpholino (MO) targeting the zebrafish FBN1 gene into Tg (fli1/EGFP) embryos. Tg embryos exhibited dilated caudal vessels, head and ocular vessels at 30–31 h after fertilization (Chen et al., 2006). In 2021, the first *fbn1* ^*+/−*^ zebrafish model was built by using CRISPR/Cas9 gene editing technology. This model was designed to mimic the genetic defects in human *FBN1*. The *fbn1* ^*+/−*^ zebrafish exhibited morphological and cardiovascular abnormalities in the juvenile stage, with marked pigmentation, increased body length, and body thinning (Yin et al., 2021). Genomic DNA was extracted from F1 embryos (hpf) 24 h after fertilization. F0 with genetic mutations were selected for mating to produce F1 progeny. f1 *fbn1* ^*+/−*^ adults were mated with Tg wild fish to produce F2 transgenic fish. f2 *fbn1* ^*+/−*^ heterozygous fish carried the mutants, which indicated that the *fbn1* ^*+/−*^ zebrafish transgenic line was successfully constructed. The first *fbn1* ^*+/−*^ zebrafish model was successfully constructed using CRISPR/Cas9 gene editing tools. This model manifested obvious morphological and cardiovascular abnormalities similar to those of MFS.

#### 5.2.5 *Caenorhabditis elegans* model of MFS

Mua-3 is the mammalian homologue of fibrillin-1 in *Caenorhabditis elegans*, the cause of MFS. Previous studies have suggested that there were possible conserved interactions between mua-3 and the TGF-β pathway in Crypto-bacterium nematodes. Crypto-bacterium cryptic rod nematodes can be used to further mimic MFS in worms (Fotopoulos et al., 2015a). In 2019, Lin *et al.* identified MFS and MFS-like mutations in the type II (LTA motifs including leucine, threonine, and alanine) receptor leading to mistransportation of the receptor through the cryptic rod nematode model of Hidradenitis elegans (Lin et al., 2019).

In MFS-like patients, three heterozygous missense mutations W521R, R528H and R537P in the TGF-β type II receptor correspond to W580R, R587H and R596P, respectively, in the cryptic nematode type II receptor DAF-4. The three-dimensional structure of the structural pairs of the c-terminal domains of the type II TGF-β receptor was investigated. The LTA motif of the kinase structural domain was exposed externally. It can interact with other proteins. The structure and function of the human type II TGF-β receptor were modelled using Pymol software. The modeling results indicate that mutations in MFS-like symptoms interfere with the structural domain located on the exposed surface of the type II TGF-β receptor. This modifies its engagement with cytoplasmic transport and/or regulatory proteins, or the activity of the receptor. This model clearly demonstrates that the function of the type II receptor carrying the MFS-like mutation is determined by somatic assays. In a wild-type background, the increase in somatotype is similar to the predominant nature of these mutations found in MFS-like patients. Cryptobacterium cryptic nematode MFS-like syndrome model provides a new paradigm for MFS-like syndrome receptor transporter-disease linkage.

#### 5.2.6 Rabbits model of MFS

Rabbits are a classic animal model species for cardiovascular diseases such as atherosclerosis, and are frequently used animal model for eye disease study.

Chen et al. (2018) truncated the C-terminal end of the *FBN1* gene by trans cytoplasmic microinjection of Cas9 mRNA and single-stranded rna (sgRNA) into fertilized embryos of rabbits using CRISPR/Cas9 technology. The heterozygous rabbit model showed muscular dystrophy, ocular syndrome, aortic dilatation and lipodystrophy in the MPL syndrome clinically (Chen et al., 2018). *FBN1* rabbits had a high mortality rate, were prone to lung infection, pneumothorax and dilated ascending aorta found in the dead rabbits. The cause of death was probably aortic root dilatation and MPL lung phenotype. The ascending aorta of *FBN1* Het rabbits was less elastic and flattened compared to the normal ascending aorta of the WT controls. In addition, the ascending and abdominal aorta were dilated in the *FBN1* Het rabbits compared to the normal ascending aorta in the WT control group. *FBN1* Het rabbits displayed skin atrophy, skin laxity and slow hair growth compared to WT rabbits. Gastrocnemius and quadriceps muscles were significantly reduced in *FBN1* Het rabbits. H&E staining and statistical analysis illustrated significant thinning of muscle fibers in *FBN1* Het rabbits. *FBN1* Het rabbit muscle atrophy, X-ray examination of the *FBN1* Het rabbit and the WT rabbit indicated that the length and diameter of the femur and tibia of the *FBN1* Het rabbit were abnormal. In addition, H&E staining demonstrated a decrease in bone marrow cells and bone marrow adipocytes in *FBN1* Het rabbits. Toluidine blue staining showed that osteoblasts were significantly reduced in the bone deposition area of *FBN1* Het rabbits. These observations indicated that *FBN1* mutations induced the typical phenotype of MPL syndrome in *FBN1* Het rabbits.

The current animal models used to study MFS are mainly based on mice. Mice reproduce quickly and in short cycles, and this model is readily available and relatively low cost (Summers, 2024). Although mouse models of MFS exhibit certain similarities to the condition in patients, they do not precisely replicate the human disease state, particularly in terms of long-term aortic dilatation and the development of coarctation (Summers, 2024). Pigs and humans share similarities in several anatomical and physiological characteristics and have body sizes suitable for a variety of surgical procedures and clinical evaluations (Jack et al., 2022a; Salinas et al., 2022). The pig model with higher maintenance costs and more resources and space are its drawbacks. Zebrafish share a highly homologous genome with humans and have a short reproductive cycle that takes up little space (Abrial et al., 2022). It makes zebrafish an ideal model for studying the molecular mechanisms and gene function of Marfan syndrome. Nevertheless, the zebrafish cardiovascular system is anatomically different from humans, which may affect research on certain cardiovascular disorders (Wang et al., 2024).

## 6 Prospectives

MFS is an inherited disorder that is rare but pathogenic. Fibrillin 1 mutations overactivate TGF-β, which leads to an imbalance in the TGF-β signaling pathway and a signaling cascade. In addition to the TGF-β signaling pathway, NO, angiotensin, WNT, NOTCH, PI3K/AKT, and other signaling pathways are disregulated in MFS. PI3K/AKT and mTOR have been associated with aortic lesion formation in MFS. However, the specific molecular mechanism of MFS still remains unclear. Treatment of MFS includes surgery and drug therapy, which is necessary for patients with high risk factors in the aorta or other organs, especially for neonatal MFS. Pharmacological treatment is a current research hotspot, and the search for new targeted drugs is predicated on the need to pass experiments with MFS model animals as well as clinical trials, and the most used model is the MFS mouse model at present. In addition to the mouse model, the rabbit model can be modeled to study the ocular phenotype of MFS, and the zebrafish model has cardiovascular characteristics to study the cardiovascular phenotype of MFS.

However, there are challenges in the research for MFS treatment. First, certain animal models exhibit unusual clinical presentations, such as no or mild AA/AC, disproportionate bone growth, lens ectasia and other clinical symptoms. Second, only part of animal genomes is available. Third, the applicability of findings from MFS animal models to the human population is constrained. Although animal models of MFS are diverse, only the rabbit model presented the symptoms of lens ectasia. In addition, researchers should consider factors such as minimal interference, reproducibility, and cost-effectiveness while conducting animal experiments. Hence, a high-quality MFS animal model should possess the following attributes: (i) obvious cardiovascular abnormalities (e.g., aortic dilatation/clamping); (ii) similarity to human MFS in skeletal malformations (e.g., curvature of spine, disproportionate growth of bones); (iii) lens ectasia similar to human MFS; and (iv) animal have homologs of human MFS-causing genes; (v) the animal model results specifically and plausibly reflect the pathologic process of human MFS.

Animal models with diverse characteristics are critical for exploring the pathophysiology and new therapeutic approaches for MFS patients. Each animal model used to simulate human MFS has pros and cons. Gene-edited animal models focus on the study of clinical manifestations, pathologic alterations, and therapeutic approaches for MFS, offering significant insights into the outward appearance and management strategies for MFS. In addition to FBN1 gene-edited animal models, other relevant gene-edited animal models are necessary to study the molecular mechanisms and gene therapies of human MFS, conjunct with novel molecular methodologies aimed at modifying the regulation of distinct genes in specific tissues in a targeted manner. Genetic models of MFS have been examined from the causative genes to the cellular and tissue levels, providing insights into the progression of MFS pathology from embryonic stages to adulthood.

The establishment of animal models of MFS can be used to probe the pathogenesis, molecular mechanisms, and pharmacological treatments of human MFS. The role of animal models in MFS is essential due to variances in anatomy, physiology, and genetics between humans and animals. These models are employed to replicate particular disease characteristics and offer significant insights to investigators. Hence, continuous endeavors have concentrated on developing a more optimized animal model that replicates the etiology and therapeutic effects associated with MFS in humans. The future trend of animal models may provide engaging insights into the comprehending of pathological and genetic characteristics, as well as novel therapeutic approaches for MFS.
